# Conservative and surgical management of small bowel diverticulitis complicated by enteroliths: A case report

**DOI:** 10.1016/j.ijscr.2025.112114

**Published:** 2025-10-27

**Authors:** Richard Lee, Jill Jozefowicz, Theodore Niemann

**Affiliations:** a Department of Surgery, 81st Medical Group at Keesler Medical Center, 301 Fisher St, Biloxi, MS, 39534, USA; b Creighton University School of Medicine, 2616 Burt St, Omaha, NE, 68178, USA

**Keywords:** Case report, Small bowel diverticulosis, Small bowel obstruction, Enterolith, Foreign body

## Abstract

**Introduction:**

Small bowel diverticulitis with enterolith-associated small bowel obstruction is a rare complication which can lead to significant morbidity and mortality. Here we present a case of small bowel diverticulitis which underwent successful conservative management at the time of initial presentation but required a surgical intervention for enterolith-associated small bowel obstruction during a subsequent hospitalization.

**Case presentation:**

We report the case of a 73-year-old female who presented with a small bowel diverticulitis and incidental discovery of an associated enterolith on contrast-enhanced computed tomography (CT). Patient responded well to nonoperative management with antibiotic therapy during the initial hospitalization. However, patient had short interval readmission due to small bowel obstruction secondary to the previously identified enterolith. Emergent surgical intervention was performed with extraction of an enterolith measuring 3 × 3 × 1.5 cm. The postoperative course was uneventful with immediate return of bowel function.

**Discussion:**

Small bowel diverticulitis with enterolith-associated small bowel obstruction has been reported as a rare complication of small bowel diverticulosis. Obstructive symptoms caused by enteroliths >2.5 cm is less likely to self-resolve with nonoperative management. Prompt surgical intervention with enterolith extraction via simple enterotomy is a safe and effective approach to prevent further complications and avoidable hospital stay.

**Conclusion:**

In patients presenting with enterolith-associated small bowel obstruction, prompt evaluation and diagnosis with contrast-enhanced CT is recommended. Large enteroliths causing obstructive symptoms should be addressed with surgical intervention.

## Highlights

• Small bowel diverticulitis with enterolith-associated small bowel obstruction is a rare complication.
• CT imaging with contrast is an effective modality for prompt evaluation and diagnosis.
• Early surgical intervention is recommended for enterolith diameter > 2.5 cm.

## 1 Introduction

Small bowel diverticula, excluding duodenal or Meckel's diverticulum, is a rare condition with a prevalence rate of 0.2 % to 1.3 % at autopsy and annual incidence ranging from 0.3 % to 2.3 %. Literatures have indicated that the prevalence typically peaks at age 50–70s and predominantly affects male patients [1]. Pathogenesis of small bowel diverticula is considered to be increased intraluminal pressure leading to herniation of mucosal and submucosal layers through weak points in muscularis layer. These pseudodiverticula occur mostly on the mesenteric border of the small bowel where the entry sites of vasa recta lead to focal weakening of the bowel wall and become more vulnerable to herniation [2]. Although often asymptomatic, small bowel diverticulosis may manifest with nonspecific gastrointestinal symptoms such as abdominal discomfort, bloating, or diarrhea. More severe presentations can include complications such as bleeding, obstruction, or diverticulitis. Diverticulitis of the jejunum or ileum may result in localized inflammation, abscess formation, perforation, or generalized peritonitis [3]. Diagnostic evaluation of small bowel diverticulitis requires a high index of suspicion, as clinical manifestations are often nonspecific and can mimic other intra-abdominal conditions. While plain abdominal radiographs are typically nonsensitive and nonspecific, cross-sectional imaging, particularly contrast-enhanced computed tomography (CT), is considered the modality of choice. CT imaging not only confirms the presence of diverticula but also allows visualization of associated inflammatory changes, such as localized bowel wall thickening, mesenteric fat stranding, extraluminal air, or fluid collections suggestive of perforation or abscess [4]. Magnetic resonance enterography/enteroclysis can be a valuable alternative in selected patients, particularly those requiring radiation-sparing modalities, although its availability and sensitivity in acute settings are more limited. Ultimately, diagnostic accuracy relies on integrating clinical findings with appropriate imaging, and in certain cases, diagnosis may only be established intraoperatively during surgical exploration prompted by suspected perforation or obstruction.

Throughout this paper, we report a rare case of complicated jejunal diverticulitis caused by enterolith impaction and subsequent partial small bowel obstruction with a review of pertinent presentation, clinical features, and management. This work has been reported in line with the SCARE 2025 criteria [5].

## 2 Case presentation

A 73-year-old female with a history of hypertension and hyperlipidemia initially presented to her primary care physician with a 2-day history of abdominal pain. CT abdomen and pelvis with IV and oral contrast was obtained which revealed a small bowel outpouching measuring approximately 5.7 × 3.0 × 2.9 cm in the left lower quadrant with surrounding inflammatory stranding**.** The diverticulum was noted to contain a linear hyperdensity with foci of air within, likely indicating mucosal enhancement or oral contrast per radiology report. No overt intraperitoneal free air, free fluid or obstructive patterns were seen (Fig. 1A, Fig. 1B). Due to the concerns for small bowel diverticulitis and contained perforation, the patient was transferred to the ED for further evaluation.

**Fig. 1A:**
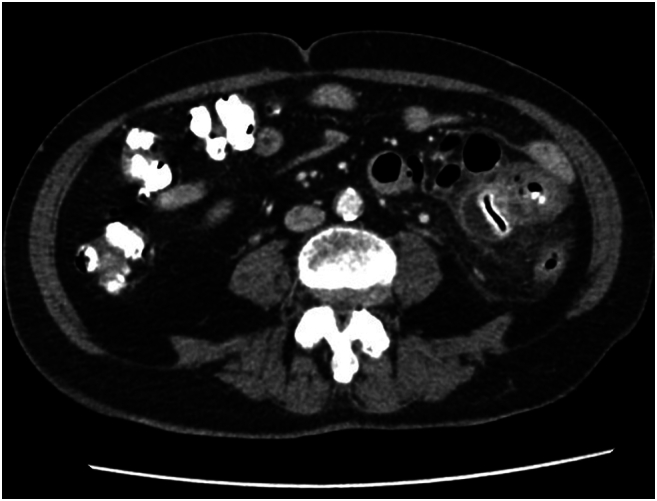
Jejunal diverticulum with enterolith.

**Fig. 1B:**
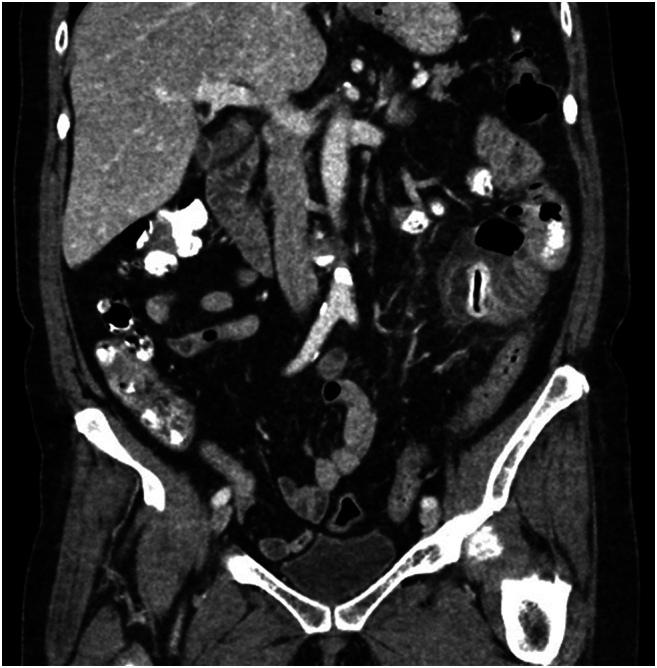
Jejunal diverticulum with enterolith.

Upon arrival to the ED, patient continued to report moderate left lower quadrant abdominal tenderness which was stable and denied any nausea or vomiting. No bowel habit changes were reported, and patient denied hematochezia or melena. Medication history consisted of Losartan 100 mg and calcium-vitamin D supplement daily. Past surgical history was significant for hysterectomy. She underwent routine screening colonoscopy approximately 6 weeks prior to the symptom onset which showed pan-diverticulosis of colon and small internal/external hemorrhoids without other concerning mass or lesions. At the time of evaluation, the patient remained hemodynamically stable and afebrile (Blood pressure 160/78 mmHg, heart rate 97 beats/min, temperature 37C, respiratory rate 18/min). Focused physical exam with focal abdominal tenderness in left lower quadrant abdomen with voluntary guarding. Laboratory test result significant for leukocytosis (WBC 18.8 10^3/uL). Remainder of serum electrolytes and liver function test values were overall unremarkable. Due to the patient's overall stable clinical presentation, a decision was made to pursue nonoperative management with fluid resuscitation, bowel rest and IV Zosyn therapy. She exhibited an uneventful hospital course with consistently improving abdominal pain, return of bowel function, adequate PO tolerance, and improving leukocytosis. On hospital day 3, her WBC normalized to 8.3 × 10^3/uL and abdominal pain had resolved. She was discharged with an additional 7-day course of amoxicillin-clavulanate 875 mg–125 mg twice a day.

Patient re-presented to the emergency department in the evening, 7 days after initial discharge due to persistent left lower quadrant abdominal pain. She was compliant to the outpatient antibiotic course. She was tolerating a bland diet at home without nausea or emesis, and continued to have regular bowel movements. No interval major health or medication changes were reported. Vital signs at the time of presentation were overall unremarkable (blood pressure 167/76 mmHg, heart rate 84 beats/min, temperature 36.9C, respiratory rate 16/min). Laboratory test results significant for WBC 16 × 10^3/uL. CT abdomen and pelvis with IV contrast was obtained, which revealed short segment enteritis with dilation, concerning for partial small bowel obstruction. Previously identified small bowel diverticulum and hyperdense foreign body were seen again. The foreign body appeared to have migrated distally in the small bowel compared to the previous imaging studies and causing proximal enteritis along the distance it travelled (Fig. 2A, Fig. 2B). She was admitted for fluid resuscitation and IV Zosyn therapy overnight. Due to the patient's persistent abdominal pain, focal tenderness to palpation on exam, leukocytosis, and failure of conservative management, a decision was made to proceed with exploratory laparotomy later that day.

**Fig. 2A:**
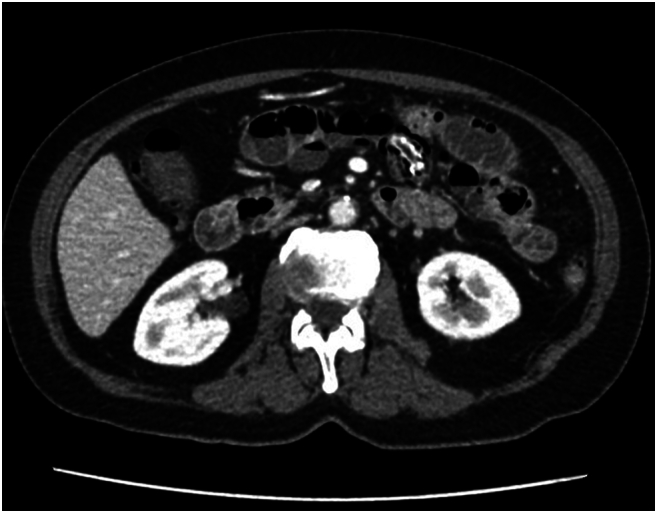
Migrated enterolith with proximal enteritis and bowel dilation.

**Fig. 2B:**
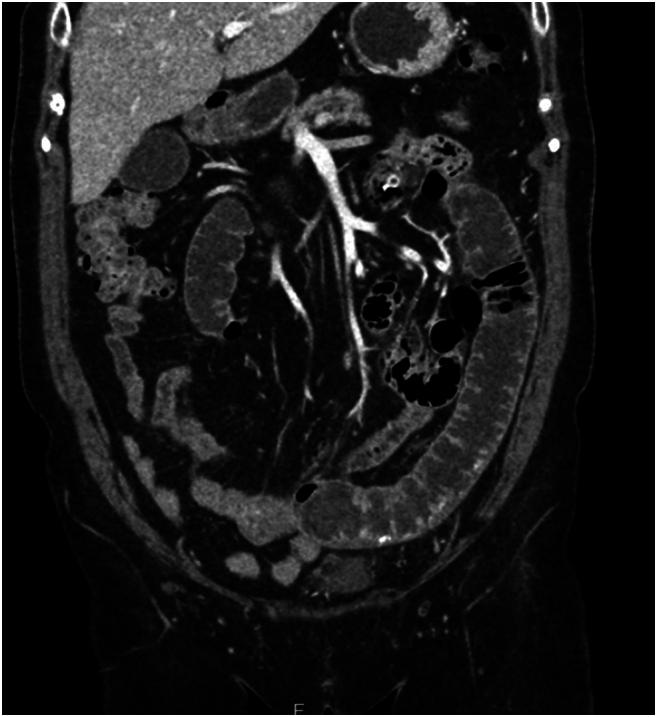
Migrated enterolith with proximal enteritis and bowel dilation.

Intraoperatively, edematous and injected proximal jejunum was noted with acute transition to normal caliber small bowel distal to the foreign object. The intraluminal object at the transition point was immobile and a longitudinal enterotomy was made on the antimesenteric border for extraction of the object. We extracted 3 × 3 × 1.5 cm circular, thickened disc-like, calcified enterolith (Fig. 3A, Fig. 3B). The enterotomy site was closed transversely in two layers, and luminal patency was confirmed. The small bowel was examined from the Ligament of Treitz to terminal ileum. A large, non-inflamed, non-perforated diverticulum was appreciated at the proximal jejunum, consistent with area of partial obstruction seen on imaging during previous admission. Surgical resection of this diverticulum was considered but its proximity to ligament of Treitz and extensive distal enteritis with associated dilation secondary to partial obstruction made it technically challenging. In the setting of no clinically significant symptoms until patient's seventh decade of life and suboptimal nutrition in the preoperative setting due to the acute disease process, we elected not to pursue further surgical intervention on the small bowel diverticulum. We closed the abdomen, and the patient tolerated the procedure well without any immediate complications.

**Fig. 3A:**
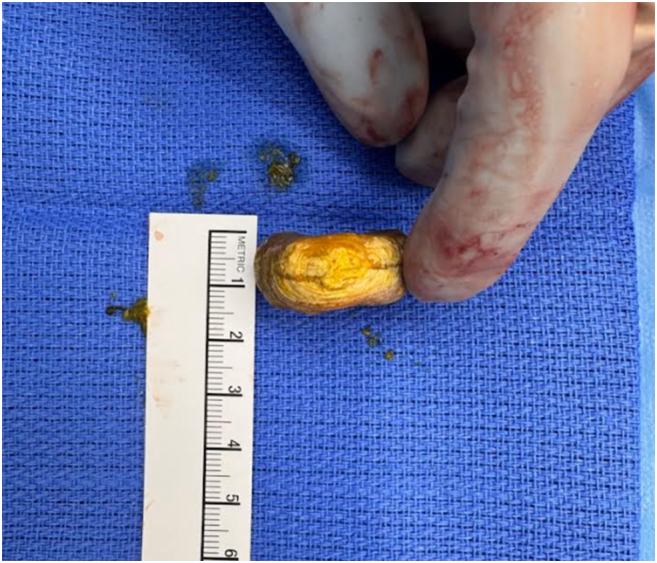
Surgically extracted enterolith.

**Fig. 3B:**
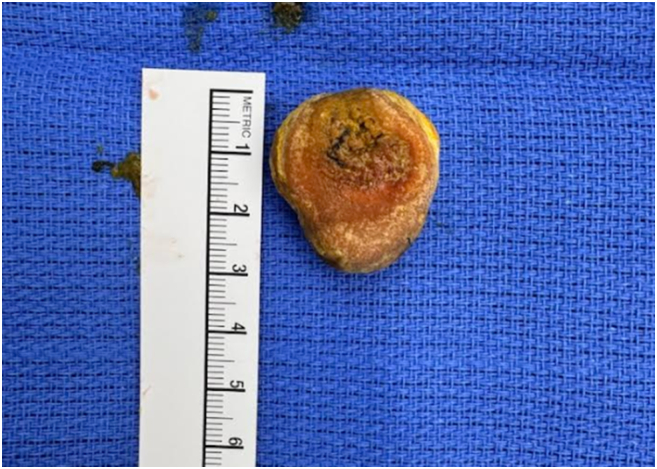
Surgically extracted enterolith.

The patient had an excellent recovery, with immediate return of bowel function and diet tolerance. She was discharged home in stable condition on postoperative day 3. Patient was evaluated again 3 weeks after being discharged with uneventful course of recovery. Patient had returned to her baseline health with regular bowel function and good oral intake without issues.

## 3 Discussion

Small bowel diverticulosis was first described in 1794 by Sommering and further explored in 1807 by Sir Astley Cooper, but no clear clinical management guidelines have been established regarding this condition due to lack of data stemming from low incidence and prevalence [6]. Due to the mild and nonspecific initial presentations of small bowel diverticulosis in most cases, its true prevalence may have been grossly underestimated. When patients present with complicated small bowel diverticulitis, it may manifest as bleeding, enterolith formation, bowel obstruction, volvulus, perforation and up to 8–30 % of these patients will require surgical interventions [1,7]. As demonstrated in this case, computed tomography may be the most effective and commonly utilized imaging modality in acutely symptomatic patients. Intravenous and oral contrast administration is helpful in diagnosis as IV contrast can reveal enhancement of bowel wall or fluid collection to detect any inflammatory processes, and oral contrast can assist with better evaluation of intra- and extraluminal abnormalities.

In this case report, we present a patient with small bowel diverticulitis and concomitant enterolith. Small bowel diverticulitis with enterolith-associated small bowel obstruction has been reported as a rare complication of small bowel diverticulosis. A review of the literature revealed 13 cases comparable to this case report with details on the size of enteroliths (Table 1). Of these, 3 reported small bowel perforation at the time of presentation [[8], [9], [10]], which required immediate surgical intervention. Of the remaining 10 non-perforated cases, 6 were managed with conservative measures initially [9,[11], [12], [13], [14], [15]] with only one patient being managed successfully with conservative measures [14]. It is important to note that the only case with successful conservative management reported an enterolith with the length of longest dimension at 2.2 cm, shortest among all the cases reviewed in this article. 10 of the 12 cases requiring surgical management underwent small bowel resection with enterolith removal [[8], [9], [10], [11],^13^,[16], [17], [18]], while two were managed with enterotomy alone [12,19].

**Table 1:** Literature review of case reports involving small bowel diverticulitis with enterolith-associated small bowel obstruction and key comparisons.

	Age/Sex	Small Bowel Diverticulitis with concomitant enterolith SBO	Attempts at conservative management	Successful conservative management	Required operative management	Small bowel resection	Enterotomy retrieval of enterolith without resection	Longest dimension of enterolith
Our Patient	73, F	X	X		X		X	3 cm
Barnard 2023 [8]	80s, M	X	X		X	X		4 cm
Chugay 2010 Case 1 [9]	79, M	X			X	X		Reported as “large”
Garnet 2011 [10]	80, M	X			X	X		2.9 cm
Gachabayov 2018 [11]	55, F	X	X		X	X		3 cm
Medsinge 2012 [12]	70, F	X	X		X		X	3.8 cm
Miranda 2023 [13]	43, F	X	X		X	X		4 cm
Sharma 2022 [14]	72, M	X	X	X				2.2 cm
Sykes 2021 [15]	79, M	X	X		X	X		4 cm
Chugay 2010 Case 2 [9]	89, F	X	X		X	X		3.5 cm
Aispuro 2019 [16]	86, M	X			X	X		3 cm
Fourneau 2018 [17]	81, M	X			X	X		3 cm
Harris 1997 [18]	56, M	X			X	X		3.5 cm
Beal 1987 [19]	73, M	X			X		X	3 cm

This case report is unique in that a single patient underwent both conservative management and surgical intervention during separate hospitalization in a short interval. During the patient's initial presentation and subsequent evaluation, nonoperative management was pursued due to no clear evidence of bowel perforation and lack of obstructive symptoms. Patient initially responded well to conservative management with antibiotic therapy, however ultimately required short interval readmission due to enteritis and recurrent partial small bowel obstruction caused by migrating enterolith. This highlights an important correlation between the diameter of the small bowel diverticulosis-associated enteroliths with likelihood of successful conservative management. As the average diameter of jejunum is noted to be approximately 2.5 cm, any enterolith diameter approaching this measure, in the setting of small bowel diverticulosis which may be caused by underlying bowel dysmotility and increased intraluminal pressure, may warrant earlier consideration for surgical intervention [20]. Surgical intervention may be pursued through either laparoscopic or open approach with the primary goal of removing the enterolith causing local inflammatory response and obstructive symptoms. Some advocate routine segmental resection of small bowel involving diverticulosis and enterolith for complete resolution of underlying anatomic abnormality. However, when small bowel resection is deemed difficult or unsafe due to various reasons such as multiple diverticula, difficult anatomic location, or diffuse bowel inflammation, a simple enterotomy with enterolith retraction may be a safe and sufficient alternative as demonstrated in this report. Careful surgical planning should take place with thorough consideration of initial patient clinical presentation and history, preoperative and intraoperative findings, and patient-surgeon discussion.

## 4 Conclusion

Small bowel diverticulitis with enterolith-associated small bowel obstruction has been reported as a rare complication of small bowel diverticulosis. In symptomatic cases, urgent CT imaging with contrast should be utilized for further evaluation and assessing the extent of the disease process. Mild symptoms with small enterolith may be amenable to conservative management. However, we recommend consideration of early surgical intervention in the setting of enterolith diameter > 2.5 cm and moderate to severe surrounding enteritis to prevent further small bowel complications and avoidable hospital utilization. Further research and additional case data on this topic will be helpful in establishing a standardized protocol for management of this disease process.

## Informed consent

Written informed consent was obtained from the patient for publication of this case report and accompanying images. A copy of the written consent is available for review by the Editor-in-Chief of this journal on request.

## Ethical approval

Ethical approval was not required for this case report in our institution.

## Guarantor

Dr. Richard Lee accepts full responsibility for the integrity of the work, had full access to the data, and controlled the decision to publish this case report.

## Methods

This work has been reported in line with the SCARE criteria.

## Registration of research studies

This study does not qualify as a First in Man study and thus does not require registration under that category.

## Funding

This research received no specific grant from any funding agency in the public, commercial, or not-for-profit sectors.

## Author contribution

Richard Lee, MD: Contributed to the study concept and literature review, participated in patient management, and reviewed and revised the manuscript critically for intellectual content. Corresponding author; involved in drafting of the initial manuscript and subsequent revisions.

Jill Jozefowicz, MD: Contributed to the study concept, supervised patient management, and reviewed and revised the manuscript critically for intellectual content. Involved in overall coordination and final approval of the submitted version.

Theodore Niemann, medical student: Assisted in literature review, and contributed to manuscript writing and formatting.

## Declaration of competing interest

There is no conflict of interest regarding the publication of this case report.
